# Network Analysis of Cattle Movement in Mato Grosso Do Sul (Brazil) and Implications for Foot-and-Mouth Disease

**DOI:** 10.3389/fvets.2020.00219

**Published:** 2020-04-29

**Authors:** Taís C. de Menezes, Ivette Luna, Sílvia H. G. de Miranda

**Affiliations:** ^1^Graduate Program in Applied Economics, Center for Advanced Studies on Applied Economics (Cepea), Luiz de Queiroz College of Agriculure (Esalq), University of São Paulo, Piracicaba, Brazil; ^2^Institute of Economics (IE), State University of Campinas (Unicamp), São Paulo, Brazil; ^3^Department of Economics, Administration and Sociology (LES), Center for Advanced Studies on Applied Economics (Cepea), Luiz de Queiroz College of Agriculure (Esalq), University of São Paulo, Piracicaba, Brazil

**Keywords:** animal movement, social network analysis, foot-and-mouth disease, potential impacts, Brazil

## Abstract

Foot-and mouth disease (FMD) is an animal disease that generates many economic impacts and sanctions on the international market. In 2018, Brazil, the world's largest beef exporter, had the recognition by World Organization for Animal Health (OIE) as a country free of FMD with vaccination and proposed to withdraw FMD vaccination throughout the country, based on a 10-year schedule, beginning in 2019. Therefore, Brazil needs studies to help the decision-making process, particularly regarding the availability of resources for strengthening of official animal health services. The state of Mato Grosso do Sul (MS) was chosen to be analyzed for three reasons: the size of its herd, the economic importance of its livestock and its location—which lies on the border with Paraguay and Bolivia. The current study adopted the Social Network Analysis and performed an exploratory analysis of cattle movement in MS. The most central municipalities in the networks were identified and they can be seen as crucial in strategies to monitor animal movement and to control outbreaks. The cattle movement networks demonstrated to be strongly connected, implying a high-speed potential FMD diffusion, in case of reintroduction. In a second stage, we performed an exploratory analysis of animal movement within the state, assuming distinct points in time for the identification of animal origin. The results of the analysis underlined the need and relevance of investing in animal control, sanitary education for producers and equipment and technologies to assist in the early detection, diagnosis, and eradication of outbreaks in a fast and efficient manner, preventing a possible outbreak from spreading to other regions.

## Introduction

Foot-and-mouth disease (FMD) is considered the most important infectious animal disease in terms of economic impact in the world (1–3). In addition to causing losses in production, it generates strong reaction from animal health systems and restrictions on animal trade inside and outside the affected country. Understanding the impacts of FMD is essential in defining the level of resources expended for its control and eradication.

Although FMD causes low mortality in infected adult animals, it causes high mortality in young animals and fetuses (4). The frequency of outbreaks and the large numbers of animals and species affected in each outbreak result in high impacts for the affected country. Outbreaks of FMD cause production losses, such as reduced milk production and contraction in livestock growth rates (1).

FMD transmission occurs through direct or indirect contact with infected animals and animal products; by humans and non-susceptible animals, vehicles and equipment that had contact with contaminated animals; as well as by soil, air and water (1). It crosses international borders through trade of infected animals and animal products. Its economic effects are amplified by imposed restrictions on international trade by importing countries (5).

The World Organization for Animal Health (OIE) endorses national programs for FMD control and provides official recognition of disease status of Member Countries, as well as establishing procedures to be followed in case of outbreaks (6). OIE orientation in crises is strategic from the economic point of view, particularly for trade, since many countries follow its guidelines to access markets in sanitary risk situations. Each type of classification leads countries to a different way of addressing the impacts of FMD, including the focus regarding research, prevention, and control (7).

In March 2017, Brazil launched a proposal to suspend vaccination against FMD throughout the country and, in May 2018, it was recognized as FMD-free with vaccination by OIE. The proposed withdraw of FMD vaccination is based on a 10-year schedule and is described in the “National Foot and Mouth Disease Prevention and Eradication Program (PNEFA): strategic plan 2017–2026” (8).

The National Foot and Mouth Disease Prevention and Eradication Program (PNEFA) was the first and largest consolidated animal health program in Brazil. The main discussions on animal health in the country and the most important health policy focus on FMD. The proposal of PNEFA 2017–2026 will promote several changes in the program and this is a topic currently under discussion.

To implement PNEFA 2017–2026, Brazil needs studies to help the decision process regarding the availability of resources to strengthen the official veterinary service, as well as to elucidate possible impacts of this structural change for the beef sector and the agencies committed to the animal health policy. The current study intends to help with this purpose.

In order to highlight the importance of animal control to prevent health crises, in particular arising from the presence of FMD outbreaks in an exporting country of the magnitude of Brazil, this paper aims to show how the movement of animals could influence the spread of an FMD outbreak. The state of Mato Grosso do Sul (MS) was selected for this analysis due to its relevance in national livestock breeding.

MS State is a major Brazilian cattle producer and supplier of calves and beef cattle to other states, representing 10% of the national herd−21 million heads (9). MS has borders with Paraguay and Bolivia—two countries in which livestock farming constitutes a relevant economic activity –, as well as with other Brazilian states that are important for the national beef cattle and dairy cattle raising (Mato Grosso, Goiás, Minas Gerais, São Paulo and Paraná). The extension of the international border enhances the risk of virus entry, and the spread from MS to other states can happen quickly, due to the significant flow of animals from this state to the rest of the country.

Beef cattle is the most important activity in the state's agribusiness gross domestic product (GDP) and it is also important for other Brazilian states. The Animal Transit Guides (Guias de Trânsito Animal—GTAs) analysis indicates that, in 2015, MS sent about 484,527 animals to other states.

The purpose of this study was to identify the flows of bovine animals between municipalities in MS by use of network analysis and to identify the most central municipalities in the network to show the importance of promoting more reinforced surveillance actions in these locations.

Epidemiological logic requires considering other susceptible animals, but this study focuses the analysis on bovine animals. The swine livestock chain is considered highly integrated in Brazil, being more organized, and more rigid in terms of health than the cattle livestock chain. Pigs move significantly less than cattle, and live a relatively shorter time. In addition, the properties that deal with pigs follow strict sanitary and sterilization protocols in their facilities. Furthermore, pig raising in the state of MS is not as strong as cattle, therefore, the second was chosen for the present study. In addition, the Brazilian buffalo herd is not significant compared to the bovine and swine herds, so the movement of animals of this species was not considered in this research.

## Materials and Methods

This study was conducted using Socioeconomic Network Analysis (SNA). In veterinary epidemiology, SNA is a statistical tool to evaluate the movements of animals that have already occurred, extrapolating to what could happen in the future. This allows evaluating the impact of disease control measures according to the network structure and predicting the potential size of the epidemic after the introduction of a highly contagious disease (10). SNA structure allows the identification of surveillance, intervention and control targets (11–26).

A network is represented by a graph, which consists of a set of vertices and lines, called nodes and links, respectively (27, 28). In this study, the nodes refer to the municipalities of MS State and the links represent the movement of animals.

In order to analyze the centrality of the municipalities within the networks, we calculate their: (i) input and output degree; (ii) weighted input and output degree; and (iii) betweenness centrality. The degree centrality measures the number of neighbors of a node. Nodes with high degree are considered hubs. In a directed network, such as an animal movement network, the indegree shows how many neighbors send animals to the analyzed node and the outdegree shows how many neighbors receive animals from the analyzed node. The weighted degree considers how many animals are sent and received, that is, it considers the thickness of the link, not the number of neighbors. The betweenness centrality is based on the idea that a node is more central if it is more important in the transmission intermediation within the network (29).

These measures show the number of nodes with which the node in question negotiates, as a receiver and as a supplier, and the node's transmission intermediation within the network. Higher levels of connectivity are indicators of vulnerability of commercial networks to infectious diseases (30, 31). The most central nodes are those whose removal would more easily interrupt the transmission process in the network (10, 32).

SNA shows that: (i) in a dense network, the animal movement is easier and faster than in a sparse network; (ii) in a disconnected network, animal movement will be slower and less embracing than in a connected network; (iii) the greater the neighborhood of a node within the network, the greater its probability of receiving the animal; (iv) a central position increases the probability of receiving the animal; (v) movement started in a central node is faster than from a peripheral node (28).

Seeking to evaluate the potential risks of FMD in Brazil, networks were built based on the registers of bovine animals circulated in the state of Mato Grosso do Sul—obtained from the Animal Transit Guides (Guias de Trânsito Animal—GTAs), which are an official control data of animal movements within and between Brazilian states—for 2015. It is worth mentioning that epidemiological logic requires considering other susceptible animals, but this study focuses the analysis on bovine animals.

The GTAs show daily records of the movement of animals within the state and provide the municipalities of origin and destination, the number of animals moved and its purpose. In the original database, these purposes were: slaughter, fattening, reproduction, sports, auctions, exports, exhibitions and service (traction). For simplification, these purposes were aggregated into four groups: slaughtering, replacement (including fattening and reproduction), events (including sports, auctions and exhibitions), and others (including exports and service^1^).

The two purposes that stand out are replacement and slaughter. It is important to differentiate between them, because animals moved for replacement still live, while animals moved to slaughter do not. Thus, the purpose of the movement can influence the process of infection transmission within the network, because animals moved to slaughtering do not continue the transmission process.

The GTA data are not published and were obtained in 2017 by the Center for Advanced Studies on Applied Economics (Centro de Estudos Avançados em Economia Aplicada—Cepea), directly with the Ministry of Agriculture, Livestock and Food Supply (MAPA)^2^, although the data are compiled by the official veterinary service of the state of MS. The database for 2015 comprised 416,743 GTA issued by the MS state. The official veterinary service has information on animal movement at farm level. However, for the accomplishment of the present study, only aggregated information at the municipal level was made available.

In addition to animal movement, data on the total number of animals of the Brazilian cattle herd were used. These data are available on the website of the Brazilian Institute of Geography and Statistics (IBGE), detailed by municipality and refer to the year 2015 to be compatible with the animal movement data (33). The Brazilian cattle herd is measured only at the end of each year and, therefore, this data is annual.

Animal movement data are recorded daily and they were analyzed on a daily basis in the construction of the networks. However, descriptive statistics on animal movements were done on monthly and quarterly basis in order to find more expressive and seasonal movement patterns throughout the year.

Daily animal movement networks were constructed. We used R software (34) to compile the animal flows with “dplyr” package (35). This package forms networks in a way that avoids networks with multiple links between two municipalities on the same day, so that the visualization of the networks was clearer—but the number of animals moved between municipalities was maintained.

For lack of more detailed data—such as the absence of data on the origin and destination of the movement in other states—the transit between states, inward and outward MS, was excluded from the analysis of the animal movement networks. The daily networks were built for the cattle movement within the state using GTA data. These networks are composed of all the municipalities of the state (*n* = 79) and the flows of animals between them.

From the networks obtained, we analyzed the dynamics of the registered flows and the descriptive statistics of socioeconomic networks for the state with “igraph” package of R software (36). Then, we evaluated the density of the daily networks and their centrality measures, showing the most central and, therefore, most vulnerable municipalities. We also generated the visualization of the daily networks for the whole year of 2015.

In the second step, also performed in R, we did an exploratory analysis of animal movement within the state, assuming distinct points in time for the identification of animal origin. This analysis aimed to show the risk of an animal contacting animals from other locations in the state, implying a potential spread of diseases—more specifically, FMD. In addition, we considered different time periods for the identification of the animal to show that time can directly affect the number of animals contacted, which consequently increases this potential transmission risk.

The exploratory analysis was based only on the animal movement data. No epidemiological simulation was performed. Thus, the results only evaluate the patterns presented by the movement of cattle. It was assumed that an animal was found in a certain municipality and that this animal left its hometown a few days before. Two scenarios were constructed: a scenario in which the animal left its hometown 3 days before discovery and a scenario in which the animal left its hometown 7 days before discovery.

After the definition of the day on which the animal was identified, the animal flows of the municipality where that animal was found were analyzed for the previous days. We did this analysis in order to calculate how many municipalities the animal could have passed during 3 days or 7 days, depending on the scenario. From the number of municipalities in which it could have passed, we calculated how many animals it could have contacted during the transit until it reached its final destination. To this end, a strong assumption was made: the animal that left its hometown could contact all animals in all the municipalities where it passed through. The number of bovine animals in each MS municipality was kept constant over the year, because Brazilian herd data are annual. For each scenario, 10,000 repetitions were performed to obtain frequency histograms. To construct these histograms, we considered all municipalities as a possible starting point and each one of the 365 days a year.

**Figure 1:**
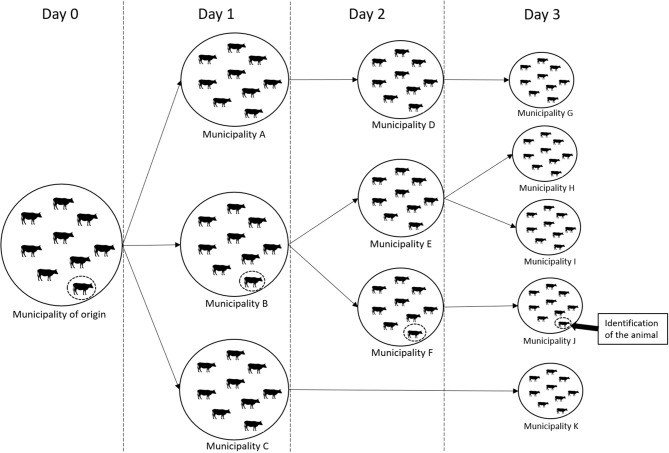
Representation of a theoretical network analysis for a 3-day scenario. Source: Authors' elaboration.

Figure 1 shows the representation of a theoretical analysis for a 3 day scenario. An animal leaves its municipality of origin on day 1 (animal circulated by the discontinuous line). Its origin could be any municipality in the state. Over the days, animals pass through some municipalities, until day 3 when an animal with clinical signs of disease is identified in municipality J. During this period, the animal could have followed different trajectories within the network. The different possible paths affect the number of municipalities it could have passed through and the number of animals, which it could have had contact to. In other words, movements of bovines happened from the municipality of origin to B, to F and to J on specific dates, and therefore the marked animal might have been moved along these links.

The histograms constructed in this stage were based on 10,000 processes as the one described above, considering the possibility of any municipality in MS as the origin and taking into account the GTAs registered in 2015. These histograms show the proportion of animals in the state that could be contacted by the animal in transit, distributed according to each iteration of the 10,000 performed.

In the third stage, we explored different cattle movement patterns from central and peripheral municipalities in the animal movement networks of MS. One municipality was selected as representative for central municipalities (Corumbá), and one for peripheral municipalities (Sete Quedas). We considered the different number of animals contacted according to the municipality of origin of the animal found. Then, it was possible to analyze how many days the animal would take on average to reach all municipalities in the state, depending on its municipality of origin.

Diffusion curves were constructed for the movement of the animal. The movement pattern is characteristic of a chain reaction in which a municipality sends the animal to one of its direct neighbors, which, in turn, send it to one of their direct neighbors in the next stage, and so on, forming a curve. These curves were constructed to emphasize that the animal movements originated in central municipalities in the network occur more quickly, offering a greater risk of spreading animal diseases to other municipalities—in comparison to the movements that begin in peripheral municipalities. We seek to demonstrate the importance of strengthening control and monitoring actions in central municipalities in order to identify risks more efficiently and quickly.

## Results

**Figure 2:**
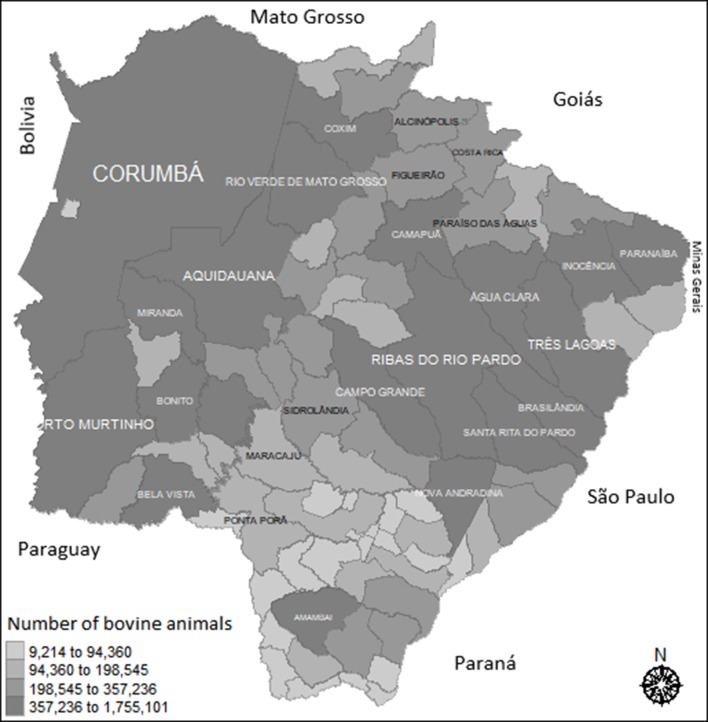
Distribution of bovine animals in Mato Grosso do Sul: 2015. Source: Elaborated from IGBE data (2016). The gray scale represents the number of animals in the municipality. Darker colors show where there are more animals.

The map in Figure 2 shows the location of MS municipalities and the size of their cattle herd, providing an overview of the geographical location and cattle distribution in the state. From the map, we observe that Corumbá is the largest municipality in the state, both territorially and in herd size and it is bordered by Bolivia; while Porto Murtinho, Bela Vista, Ponta Porã and other municipalities are located on the border with Paraguay. The international border area and the area near the border with the states of São Paulo and Minas Gerais (Paranaíba, Água Clara, Três Lagoas, Ribas do Rio Pardo) represent the majority of the cattle herd of MS state.

### Characterization of Animal Movements

**Figure 3:**
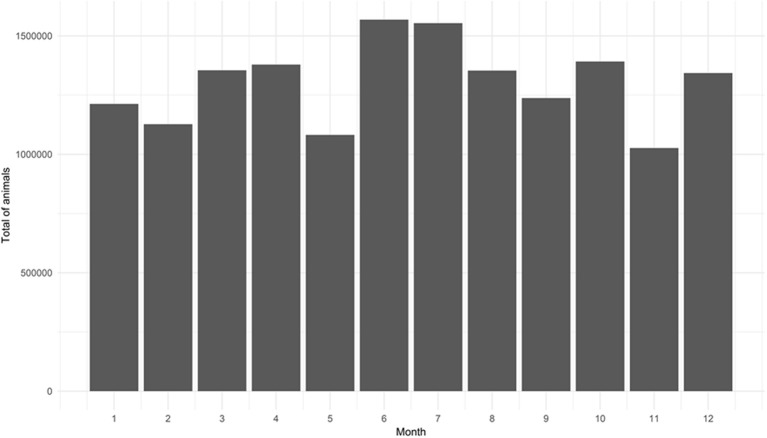
Monthly total of animals moved in 2015 according to Animal Transit Guides (GTAs) issued in Mato Grosso do Sul. Source: Elaborated from GTAs.

In 2015, 12.35 million bovine animals were moved within MS, of which 65% were for replacement, while 30.3% were for slaughter and 4.7% for events; only 95 animals were moved for other purposes. The number of animals moved between the municipalities of MS varied across the months, not only in the number of registered GTAs (number of transactions) but also in terms of animals moved monthly, with the most movements in June and July and the fewest in November (Figure 3). In general, the predominant purposes in animal movements were slaughter and replacement, distributed throughout the quarters (Table 1).

**Table 1:** Total cattle sent for slaughter and replacement in MS: 2015.

**Period**	**Slaughter (in millions)**	**Replacement (in millions)**
1st quarter (January–March)	1,04	1,75
2nd quarter (April–June)	0,93	2,16
3rd quarter (July–September)	0,88	2,23
4th quarter (October–December)	0,89	1,88

*Source: Elaborated from GTAs*.

The 10 municipalities with the largest herds of the state are: Aquidauana, Camapuã, Campo Grande, Corumbá, Coxim, Porto Murtinho, Ribas do Rio Pardo, Rio Verde de Mato Grosso, Santa Rita do Pardo, and Três Lagoas. These municipalities were among the top 10 recipients and senders of animals within the state.

In the same year, MS remained a net exporter of cattle, sending additional 484,527 animals to 21 other states and receiving 280,421 animals from other Brazilian states. Nearly 58% of the total sent was intended for slaughter, while only two animals were received for this purpose. Among animals transported to other states, 42% were destined for replacement; while that purpose accounted for about 92% of the total received. The main recipient of animals for slaughter and replacement was the state of São Paulo, while Minas Gerais was the main supplier of animals for replacement to MS.

During 2015, the 10 municipalities that received the most animals for slaughter purposes concentrated a significant portion of the total transported volume−73.9%. These municipalities accounted for <14% of the state cattle herd. Considering the animals sent for slaughter, the flows were less concentrated, with the top 10 municipalities accounting for <33% of the total animals moved with this purpose and more than 32% of the total state cattle herd.

The 10 municipalities that received the most animals for replacement purposes concentrated 31% of the total flows. These municipalities accounted for almost 35% of the state cattle herd. Considering the animals sent for replacement, the top 10 municipalities accounted for 40% of the total animals moved with this purpose, concentrating about 35% of the state herd.

### Daily Networks of Animal Movement in Mato Grosso Do Sul

**Figure 4:**
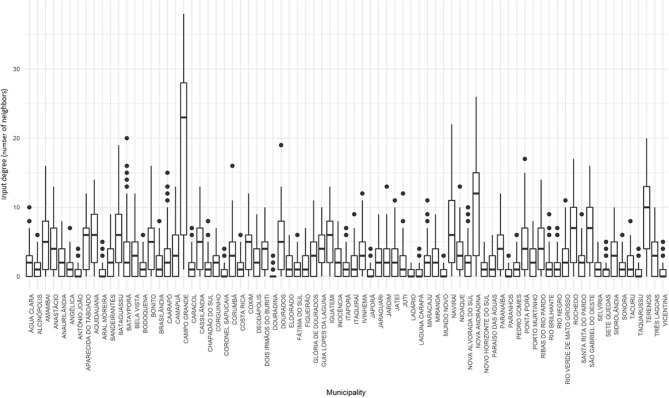
Input degree for cattle movement in Mato Grosso do Sul: 2015. Source: Elaborated from GTAs. Number of daily partners of each municipality distributed in quartiles (boxplots).

A network was built for each day of 2015 and the three proposed indicators of centrality were calculated for all municipalities of the state. For the degree centrality, Campo Grande, Naviraí, Nova Andradina and Terenos showed the highest input degree, which means that there was a greater local density in their neighborhood, since there was a greater number of direct neighbors (or municipalities), acting as the main receivers of animals (Figure 4). Among them, Campo Grande stood out for having a median of direct neighbors superior to that of the other municipalities (above 20).

We can also notice that, for Campo Grande, the variability was also high when compared to the observed in the other municipalities. Thus, median and values observed in the last quartile were significantly higher than those for other municipalities. This can indicate that Campo Grande is, on average, more vulnerable to an FMD outbreak, since it receives animals from many different municipalities, presenting a higher probability of receiving infected animals in the event of an outbreak.

**Figure 5:**
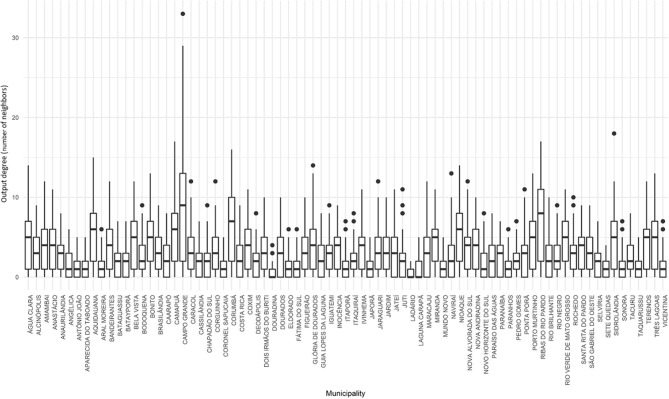
Output degree for cattle movement in Mato Grosso do Sul: 2015. Source: Elaborated from GTAs. Number of daily partners of each municipality distributed in quartiles (boxplots).

On the other hand, the output degree average values were not so heterogeneous (Figure 5). These values showed a dispersion among the municipalities, but they were not as discrepant when compared to the input degree. In this case, Aquidauana, Campo Grande, Corumbá and Ribas do Rio Pardo stood out, presenting medium values >15 direct neighbors—although Campo Grande was still most extreme. In other words, these municipalities acted as the main animal suppliers within the network, as they sent to a greater variety of municipalities. In epidemiological terms, they could function as major spreaders of the FMD virus, in case of infection—spreading it to an expressive portion of the state.

**Figure 6:**
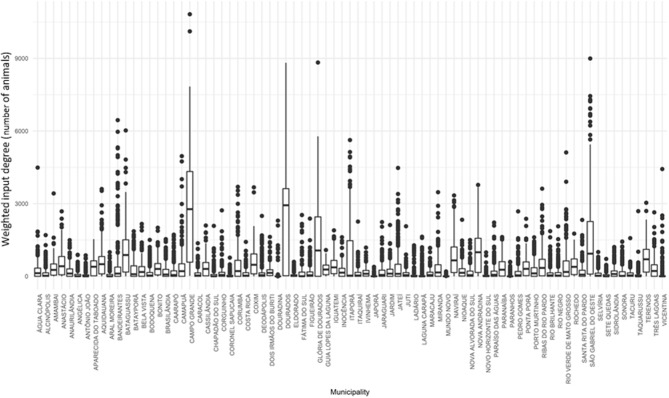
Weighted input degree for cattle movement in Mato Grosso do Sul: 2015. Source: Elaborated from GTAs. Number of animals sent daily to each municipality distributed in quartiles (boxplots).

In the case of the weighted input degree (Figure 6), the municipalities that stood out for receiving more animals, in median values, were Campo Grande, Dourados, Glória de Dourados and São Gabriel do Oeste. The quantity of atypical values^3^ was also highlighted in this figure, where each of the points outside the boxplot represents a specific day in which there was an extreme movement (in number of animals) when compared to the values predominant in the sample. These municipalities, therefore, would be more vulnerable to an outbreak of FMD because they receive more animals than the rest of the state.

**Figure 7:**
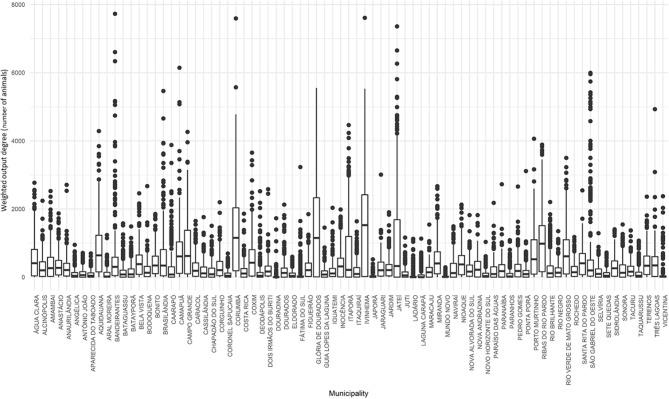
Weighted output degree for cattle movement in Mato Grosso do Sul: 2015. Source: Elaborated from GTAs. Number of animals sent daily by each municipality distributed in quartiles (boxplots).

Corumbá, Glória de Dourados, Ivinhema, Jateí and Ribas do Rio Pardo stood out in terms of weighted output degree (Figure 7). Again, there was a significant number of outliers in the distributions for each municipality. These central nodes, according to this measure of centrality, also constitute potential large spreaders of many infectious animal diseases, including FMD virus, by sending large numbers of animals to other municipalities within the state.

**Figure 8:**
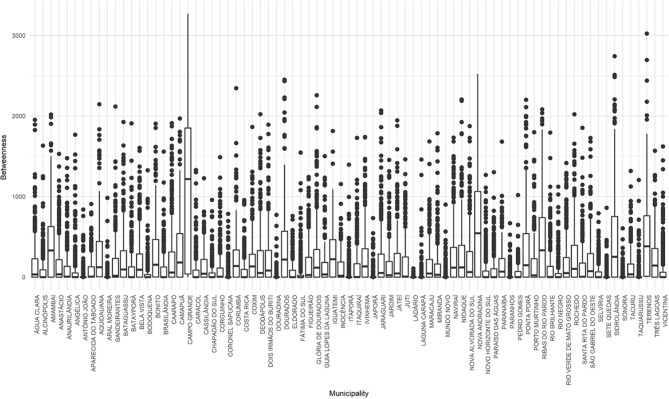
Betweenness centrality for cattle movement in Mato Grosso do Sul: 2015. Source: Elaborated from GTAs. Number of relations intermediated daily by each municipality distributed in quartiles (boxplots).

The betweenness centrality showed which nodes would cause the reduction of the network connectivity if they were removed, thus slowing down the transmission process. Campo Grande, Nova Andradina, Ribas do Rio Pardo and Terenos were the nodes that played a major role in the intermediation of flows between the different municipalities of the network (Figure 8). Finding these nodes with high intermediation helps to understand who can control the animal flows from one part of the network to another. The removal of these nodes fragments in the network could make it easier to control a sanitary crisis in a possible scenario of FMD reintroduction.

**Figure 9:**
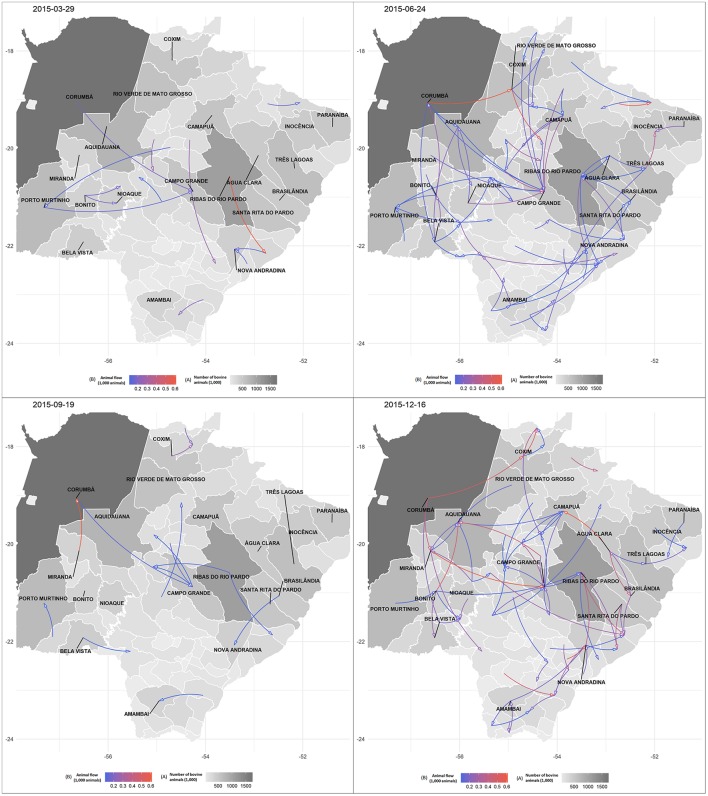
Representation of four daily cattle movement networks in Mato Grosso do Sul: 2015. Source: Elaborated from GTAs and (33). **(A)** The gray scale represents the number of animals in the municipality. Darker colors show where there are more animals. **(B)** The red scale shows the number of animals moved between municipalities on that date. Blue denotes small number of animals. Purple represents a relatively average number of animals. Red indicates a large number of animals.

Figure 9 shows a representative view of the daily networks analyzed for 2015, for 4 days of the year (randomly selected by R), showing the most intense flows in the red color and the size of the herd in the gray scale. In some days of the year, the flow of animals between the municipalities is intense, while in other days this flow is reduced, with movement of few animals between neighboring municipalities. When examining the networks for every single day of 2015, in general and regardless of the month analyzed, there was a process of “supply” in the municipalities, which preceded more intense movements between central municipalities.

### Exploratory Analysis of Animal Movement in Mato Grosso Do Sul

**Figure 10:**
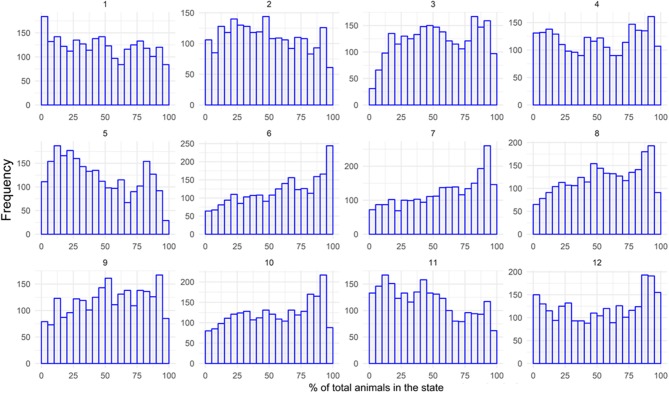
Frequency histograms showing the numbers of iterations out of 10,000 (y-axis) which end with a certain percentage of animals contacted (x-axis) per month in Mato Grosso do Sul: 3 days of lag. Source: Elaborated from GTAs.

The first scenario assumed that a bovine animal was identified in a certain municipality 3 days after leaving its hometown. Figure 10 shows the frequency histogram of the proportion of animals from the MS herd that could have contact with that animal.

January (1) and June (6), for example, had different potential impacts. In January, the repetitions were distributed almost uniformly over the proportion of the herd possibly contacted, with the vertical axis representing the number of repetitions that resulted in a certain affected proportion of the herd (x-axis). On the other hand, in June, many repetitions indicated a significant share of the herd that could be contacted by the animal, and many of them resulted in an impact on 100% of the MS herd.

In general, there was a great dispersion in the distribution of this impact, with a possibility of contact varying from 0 to 100%, depending on the animal's origin. This is because movements started in central nodes can be faster than those from peripheral nodes (with few direct neighbors and less significant animal flows).

The same exercise was repeated considering the distribution of the total number of municipalities that the animal could reach. The number of potentially contacted municipalities for a 3 day lag varied between 1 and 79 (total municipalities in the state), depending on the origin of the animal.

**Figure 11:**
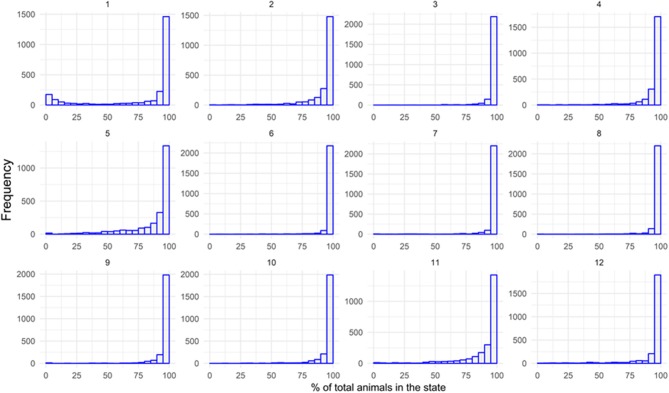
Frequency histograms showing the numbers of iterations out of 10,000 (y-axis) which end with a certain percentage of animals contacted (x-axis) per month in Mato Grosso do Sul: 7 days of lag. Source: Elaborated from GTAs.

The second scenario considered that it took 7 days to identify the animal. The results were even closer to the upper limit (more extreme). Although there was dispersion in the distribution of impact on the herd (it could reach one animal or the entire herd), the probability of contact between the animal and the rest of the herd changed to a higher interval, between 75 and 100%, according to the distribution estimated (Figure 11). The number of reached municipalities also varied less between 1 and 79, concentrating between 60 and 79 municipalities. In other words, the probability of reaching most of the states' municipalities (or even all of them) was higher.

### Differences Given by the Starting Point (Central and Peripheral Municipalities)

After studying the centrality measures of all the built networks, two municipalities (out of 79) were selected as starting point for animal movements, Corumbá as an example of a municipality with high centrality in the network, and Sete Quedas as an example of a peripheral municipality. In addition to appearing as one of the most central cities, Corumbá has the second largest cattle herd in the country, it is located on the border between Brazil and Bolivia, and, hence, is a high-risk municipality in terms of animals' entry (legal and illegal) by the border. Sete Quedas did not play a central role in the state's cattle movement networks, presenting considerably smaller animal flows than Corumbá.

**Figure 12:**
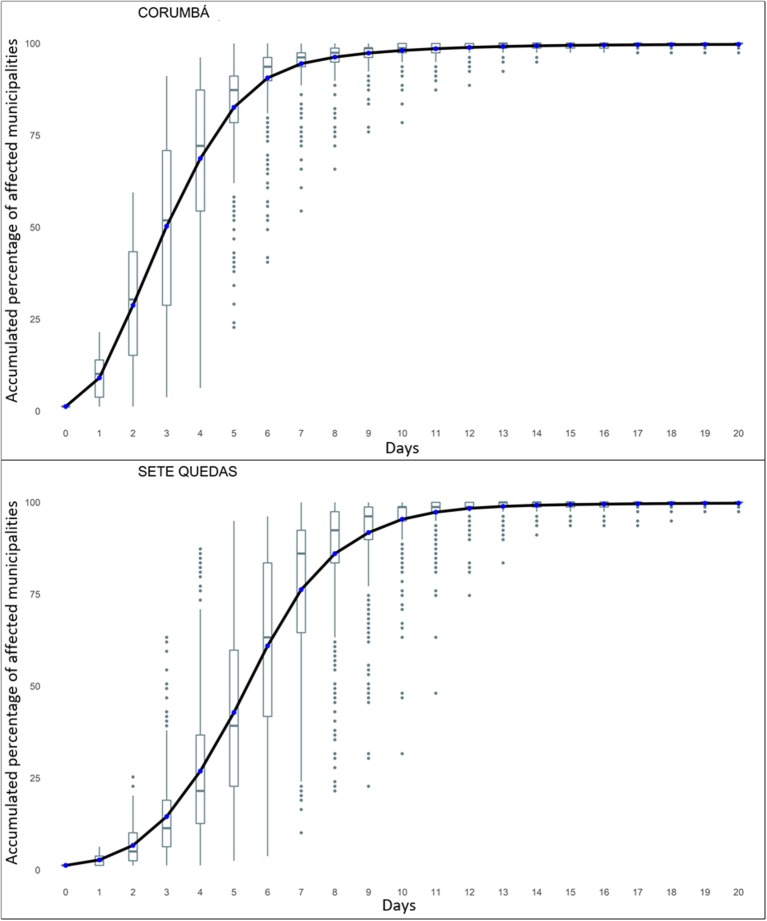
Diffusion curves for animal movements within Mato Grosso do Sul initiated in Corumbá and Sete Quedas. Source: Elaborated from GTAs. Note: Number of affected municipalities per day distributed in quartiles (boxplot). The blue dots represent the average of the distributions and were interconnected to form the accumulated average curves.

This comparison demonstrated how different the movement speed was when the process originated from a central node and when, alternatively, it did from a peripheral node. In Figure 12 it is possible to compare transmission processes with different starting points: Corumbá and Sete Quedas. The x-axis shows the number of days during which the animal could move and the y-axis shows the proportion of municipalities in the state that the animal could pass during the days of movement. The figure shows average cumulative diffusion curves for 2015, determined by the daily distributions of movements initiated in the two chosen municipalities. The points outside the curve are outliers observed throughout the year.

Both movement paths occurred in an accelerated way. However, the process started in Corumbá occurred faster than in Sete Quedas, as the former showed a steeper average curve compared to the second, underlining the high speed of animal movements started in a central node. If the animal originally left Corumbá, it would take 10 days to reach 100% of the state's municipalities on average. Whereas, if it departed from Sete Quedas, that period would increase to 14 days on average.

However, there was a notable dispersion, mainly, in the initial stage of the movement path—evidenced by outliers. Therefore, when considering the daily dispersion curves—instead of just the average curve—it could be possible to find S-shape curves; underscoring differences in movement velocity according to the observed day. It is important to note that these curves represent only the number of municipalities that the animal could pass through, not taking into account the number of animals that could have contact with it.

The fact that the mean curves did not have a S-shape can be a sign that the movement path in the network was very fast, even for the peripheral municipality (in the network) of Sete Quedas. This shows that, on average, the structure of the MS animal movement networks was very connected, which could accelerate the speed of FMD propagation within the state, amplifying the outbreaks' impacts.

## Discussion

Geographical distribution of susceptible animals is among the factors influencing the spread of FMD virus. Consequently, it is important to identify the municipalities or areas where there is a high concentration of animals. In case of an infection with FMD virus, the greater the number of susceptible animals, the greater the risk of transmission between them. Municipalities or areas with high dense farms or animals are at the high risk for disease incursion, therefore, these areas should have intensive surveillance system in place.

The large number of FMD susceptible animals in the international border raises the probability of infection by any infected animals crossing from one country to another. The international border region of MS stands out as the most vulnerable to failures of inspection or possible illegal movements and should receive increased attention during surveillance actions—because there is a possibility of entry of susceptible animals from other countries in more than 600 kilometers of dry frontier.

In addition to the fact that surveillance at the international border is not 100% effective, the most recent FMD outbreaks in the region occurred in areas that were recognized as being free of FMD with vaccination (MS in 2005/2006 and Paraguay in 2011) and these outbreaks origin is still unknown (37, 38).

Amaral et al. (2) and Santos et al. (39) show that the international border of MS with Paraguay and the international border of Rio Grande do Sul (another Brazilian state) with Argentina and Uruguay are factors that represent a high risk of reintroduction of the disease by illegal or informal animal movements between countries. Therefore, it is important to consider the international border as a risk factor.

Animal movement is an important factor for the spread of FMD. MS can be in a situation of greater vulnerability regarding the introduction and spread of FMD virus by receiving many animals for replacement, predominantly. This is because animals for replacement coming from other states could be infected, in case of virus reintroduction in Brazil, and would remain alive when they entered the properties of MS—presenting a risk of virus transmission within the state herd.

For lack of more detailed data and for simplification purposes, the transit between states, inward and outward the MS, was excluded from the analysis of the animal movement networks. However, regarding epidemiological risk issues, it is important to emphasize the state's importance as an animal supplier to the rest of the country as well as a recipient of animals from other states—especially for epidemiological risk issues.

In case of reintroduction in MS, more intense movements of bovine animals, like in June and July, may result in a greater risk of spreading the FMD virus. Therefore, it can be inferred that surveillance should be reinforced in this period, from the perspective of preventive planning for animal health policy. On the other hand, May and November showed a significant fall in the animal movements. The most likely cause is vaccination campaigns against FMD. During this period, producers gather their animals to apply the vaccine and there is less movement between properties. Therefore, the risk of transmission within the network in these months could be slightly lower than in the rest of the year.

The quarterly analysis of animal flows may reveal relevant elements related to the systems of production and their cycle, in terms of birth, weaning, fattening, and slaughter. Seasonality, inferred by the observation of the quarterly data, relates to the dry season, the confinement period and the usual time of commercialization of these confined animals, of calf birth and weaning age. Seasonal trends and temporal variation of animal movement are not uncommon in livestock networks already built for several other countries (18, 20–22, 24, 31).

In the first quarter, the slaughter rate was higher than in the other quarters; this is due to the precedence regarding the drought period. In the following quarter, when the drought season begins, the number of animals moved for slaughtering and replacement increased. In addition, in May, the weaning process of calves begins, which also justifies the increase of animals moved for replacement in the second and third quarters.

Cattle breeding has a certain seasonality throughout the year, divided in two periods: favorable and unfavorable. In the case of a tropical climate, as observed in the Center-West of Brazil, spring and summer (from September to February) are favorable for livestock, since they characterize the rainy season, improving the growth of the pastures. On the other hand, autumn and winter (from March to August) are unfavorable to the activity because the lack of rain causes the pasture to dry, thus reducing the food supply for the herd. This implies a higher slaughter rate, usually before the drought period, and, consequently increased animal movement during those seasons, justifying the greater movement observed during June and July.

If the FMD virus found its way to a highly-connected node on the network, many municipalities could become infected before authorities were aware of the virus circulation. This could hamper the control of the infection spread by the authorities, and lead to an outbreak explosion, depending on the level of the herd immunization. Immunized animals would spread the virus slower than in an unvaccinated population.

Regarding animal movement networks, we observed that measures of animal entry had greater heterogeneity than measures of animal exit, which has already been observed in animal movement networks in Argentina (20), France (40), and United Kingdom (19).

In general, the connectivity distributions of the network nodes were distorted. Most nodes had only a few connections and a small minority of them had many connections. In some cases, the mean levels of connectivity were higher than the interquartile range of the data, highlighting the presence of outliers. In addition, the municipalities with the largest number of direct neighbors (considering input and output) were those that moved the largest number of animals, generally acting as major suppliers or receivers of animals. Campo Grande, for example, has a large movement of animals, consisting of a central node in the state's cattle movement networks. This central role may be due to the large slaughtering facilities located in this municipality.

Centrality measures pointed to the same municipalities, so they function as hubs of movement, with many connections within the networks. This means that the most central nodes in the networks are vulnerable in different ways and have a greater potential to infect a large part of the network in a possible outbreak. Therefore, during an outbreak, targeted surveillance for central municipalities located in unaffected regions may have a greater probability of detecting the virus introduction into new areas. Likewise, movement restrictions directed at such municipalities may be more likely to prevent a wider spatial spread of the disease (24).

It is noteworthy, however, that this analysis considers only the movement of animals within MS State. If interstate animal movement were considered, the classification of central and peripheral municipalities could change, highlighting the importance of municipalities that in this study were classified as peripheral.

Animal movements were predominantly local (between geographically neighboring municipalities), although large municipalities were more likely to be involved in long distance movements (crossing the state). There was movement of animals throughout the whole state. However, movements of large quantities of animals were mostly local, between geographically close municipalities. This is important to notice because the frequency of animals moving over long distances is a factor that increases the risk of FMD infection and spread in the eventual reintroduction of the virus in the state.

The exploratory analysis based on the identification of an animal in a given municipality aimed to show the potential number of contacts with other animals along the trajectory of the identified animal. In this sense, the difference between the scenarios results shows that identification in a shorter period of time can significantly reduce the likelihood of contact between animals. In the context of an FMD outbreak, this analysis could indicate that the faster identification of animals that have moved from their origin reduces the probability of spreading the virus.

The analysis also shows that the transmission process is faster from a central municipality in the animal movement network, in comparison to a process initiated in a peripheral. This reinforces the importance of promoting more reinforced surveillance and control measures in central municipalities, as they could function as large hubs for the spread of the FMD virus in case of reintroduction in the state. The results show signs that FMD outbreaks originating in central municipalities could cause more damage than outbreaks started in peripheral municipalities within the state. This analysis could be expanded if the movement of animals between MS and other Brazilian states was considered. It would be possible to analyze the potential for spreading the virus at the national level.

In order to carry out the exploratory analysis, a very strong assumption was made: the animal in transit could have contact with all animals wherever it passed. This ended up overestimating the percentage of the state herd that could have contact with this animal. In a study with more detailed data, it would be possible to better consider this contact rate, in order to bring the results closer to reality.

The Brazilian official veterinary service has information on animal movement at farm level. However, for the accomplishment of the present study, only aggregated information at the municipal level for 2015 was made available. The request to access the most recent data to update the study was denied, as well as access to detailed data.

This was a limitation that directly affected the results of the study. The analysis of centrality measures at the property level could reveal properties with a central role in animal movement networks, even though they are located in peripheral municipalities. This could assist in defining more efficient and effective surveillance and control measures than measures based solely on data analysis at the municipal level.

In addition, the exploratory analysis would be completely different, because it would consider a level of aggregation of the number of animals considerably lower than the total number of animals in a municipality. This would directly affect the proportion of the herd that could be affected by the animal in transit. It would also dramatically alter the diffusion curves, making them significantly smoother. It would take a much longer period of time to reach 100% of the state's municipalities, as animal flows would be much more dispersed. This is the major limitation of the current study, caused by the lack of access to more detailed data.

It is known that official databases do not cover 100% of the flow of animals within the country. In fact, there are studies that seek to estimate how much animal movement occurs outside the official records, like Correr et al. (41), who estimated that almost 10% of cattle traffic in Brazil has no official record. However, there are no means to track such movement nowadays and, therefore, the present study considered only the official data to construct the movement networks.

Papers in the veterinary field are usually based on farm-level data, with geographic information. The unavailability (for this study) of more disaggregated and geo-referenced data did not allow more complex analyzes. This reinforces the need for investments in animal movement control and in tools that allow easy localization of livestock within Brazilian territory—improving the capacity to respond to sanitary emergencies, and to reduce the impacts of eventual outbreaks.

In Brazil, this is particularly relevant because the country intends to submit its candidature to the status of free of FMD without vaccination and to do that, it will be necessary to phase out the vaccination campaigns in the next few years. The possible impacts of a FMD reintroduction in the country would certainly be more dramatic if the herd is not immunized anymore—given that in an important state in cattle farming, such as MS, the animal movement networks are strongly connected and present municipalities with significant animal flows.

Thus, it is essential to assure that, before removing the vaccination, the states' animal health services as well as the federal service are ready to enhance the level of security by inspection and monitoring as well as by an efficient system of intelligence that would allow for a quick response in case of an outbreak. For that, it is important to consider municipalities (or farms) that concentrate many animals and animal flows—those that are more central in the movement networks.

Other factors can be considered, so we can build a model for the disease transmission in Brazil, such as: number of animals transported; number of animals passing through the border without inspection; other susceptible species; movement of animal products; FMD transmission rate; effective vaccination rate; effectiveness of sanitary inspection, inside and outside the farms. Ideally, Brazil should have at least one model for the FMD virus spread, such as AusSpread in Australia (42) and NAASDM in United States (43), to assist in the formulation of public policies, allocation of resources, and development of an outbreak response plan. In the meantime, studies like this seek to promote discussions and assist policy makers in order to contribute to the development of Brazilian livestock.

## Conclusions

This characterization and exploratory analysis of cattle movement between municipalities of Mato Grosso do Sul State (MS, Brazil) identifies the regions and periods of higher animal flow density and, therefore, periods in which all municipalities of the state are in a situation of greater vulnerability.

Cattle movement networks within the state demonstrated to strongly connect municipalities. This implies a high-speed potential of FMD transmission in the state. As MS sends animals to other Brazilian states, the outbreak could spread to other locations in the country. The greater the infected area, the greater the economic impacts of the disease, which include everything from control and containment of the outbreak, to market losses and reduction in beef prices—impacting the whole livestock chain and all the other sectors of the economy interconnected with it.

The analysis demonstrates the need and importance of investing in animal control, sanitary education for producers and equipment and technologies to assist in the early detection, diagnosis, and eradication of outbreaks in a fast and efficient manner, preventing a possible outbreak from spreading to other regions.

The scarcity of studies on this subject makes this exercise an initial step toward further developments in order to explore a matter of such importance for the state and for the Brazilian economy as a whole. In future research, machine learning and big data tools could be exploited to improve the analysis, in order to generate scenarios as tools for police markers. All limitations of this work were conveyed to the decision makers at the end of the project.

Despite the history of crises and the significant portion of domestic exports affected, the economic impacts of FMD in Brazil are still poorly understood. Further studies, based on detailed data and the application of robust economic models, based upon epidemiological models, should be promoted in order to accurately measure the risks and impacts of the disease in the country, and thus improve decision-making regarding sanitary actions and animal health protection.

## Data Availability Statement

The raw data supporting the conclusions of this article will be made available by the authors, without undue reservation, to any qualified researcher.

## Author Contributions

TM was guided by the professors during the master's degree and this article is derived from the dissertation. SM contributed to the analyzes of Brazilian livestock and foot-and-mouth disease. IL contributed to the methodology of analysis of socioeconomic networks.

## Conflict of Interest

The authors declare that the research was conducted in the absence of any commercial or financial relationships that could be construed as a potential conflict of interest.

## Footnotes

^1^There is no significant movement of animals for service and, when such movement exists, animals generally do not travel long distances nor are they sent to several other properties. Therefore, this purpose was grouped together with exports.

^2^GTA data is confidential and belongs to the Brazilian Ministry of Agriculture Livestock and Food Supply. Access to the most recent data for updating the study before publication was not authorized.

^3^The atypical values (outliers) here are the observed values that were outside the upper quartile.

## References

[1] Knight-Jones TJD Rushton J. The economic impacts of foot and mouth disease: what are they, how big are they and where do they occur? Prevent Vet Med. (2013) 112:161–73. 10.1016/j.prevetmed.2013.07.013 23958457 PMC3989032

[2] Amaral TB Gond V Tran A Mapping the likelihood of foot-and-mouth disease introduction along the border between Brazil and Paraguay. Pesquisa Agropecuária Brasileira. (2016) 51:661–70. 10.1590/S0100-204X2016000500029

[3] Feng S Patton M Davis J. Market impact of foot-and-mouth disease control strategies: a UK case study. Front Vet Sci. (2017) 4:129. 10.3389/fvets.2017.00129 28920059 PMC5585142

[4] Ranjan R Biswal JK Subramanianm S Singh KP Stenfeldt C Rodriguez LL. Foot-and-mouth disease virus-associated abortion and vertical transmission following acute infection in cattle under natural conditions. PLoS ONE. (2016) 11:e0167163. 10.1371/journal.pone.0167163 27977708 PMC5157973

[5] Miranda SHG Evaluating WTO institutions for resolving trade disputes involving non-tariff measures: four cases involving Brazil. In: Brouwer F, Fox G, Jongeneel, R, editors. The Economics of Regulation in Agriculture: Compliance with Public and Private Standards, Oxfordshire: CAB International (2012).

[6] OIE World Organisation for Animal Health Terrestrial Animal Health Code. Paris (2016). Available online at: http://www.oie.int/international-standard-setting/terrestrial-code/access-online/ (accessed in Jan. 11, 2017).

[7] Knight-Jones TJD Mclaws M Rushton J Global foot-and-mouth disease research update and gap analysis. Transboundary Emerg Dis. (2016) 63:42 10.1111/tbed.12523 27363718

[8] MAPA (Ministério da Agricultura Pecuária e Abastecimento) (2017). Plano Estratégico do Programa Nacional de Febre Aftosa 2017–2026. Brasília. Available online at: http://www.agricultura.gov.br/assuntos/sanidade-animal-e-vegetal/saude-animal/programas-de-saude-animal/febre-aftosa/pnefa-2017-2026/arquivos/1_pnefa_-_plano_estrategico_2017_2026_gt_mar_29_v3.pdf (accessed Jun. 15, 2017).

[9] IBGE (Instituto Brasileiro de Geografia e Estatística) (2017). Pesquisa Trimestral do Abate de Animais. Available online at: https://sidra.ibge.gov.br/home/abate/mato-grosso-do-sul (accessed Jun. 27, 2017).

[10] Dubé C Ribble C Kelton D Macnab B. Introduction to network analysis and its implications for animal disease modelling. Revue Sci et Tech. (2011) 30:425–36. 10.20506/rst.30.2.2043 21961215

[11] Webb CR Sauter-Louis C Investigations into the Contact Structure of the British Sheep Population. In: Meeting of the Society for Veterinary Epidemiology and Preventive Medicine. Cambridge (2002). p. 10–20.

[12] Green DM Kiss IZ Kao RR. Modelling the initial spread of foot-and-mouth disease through animal movements. Proc R Soc London B. (2006) 273:2729–35. 10.1098/rspb.2006.3648 17015320 PMC1635508

[13] Kiss IZ Green DM Kao RR. The network of sheep movements within Great Britain: network properties and their implications for infectious disease spread. J R Soc Interf. (2006) 3:669–77. 10.1098/rsif.2006.0129 16971335 PMC1664651

[14] Ortiz-Pelaez A Pfeffer DU Soares-Magalhães RJ Guitian FJ. Use of social network analysis to characterize the pattern of animal movements in the initial phases of the 2001 foot mouth disease (FMD) epidemic in the UK. Prevent Vet Med. (2006)76:40–55. 10.1016/j.prevetmed.2006.04.007 16769142

[15] Webb CR Investigating the potential spread of infectious diseases of sheep via agricultural shows in Great Britain. Epidemiol Infect. (2006) 13:431–40. 10.1017/S095026880500467X PMC2870361 16409648

[16] Kao RR Green DM Johnson J Kiss IZ. Disease dynamics over very different time-scales: foot-and-mouth disease scrapie on the network of livestock movements in the UK. J R Soc Interf. (2007) 4:907–16. 10.1098/rsif.2007.1129 17698478 PMC1975769

[17] Robinson SE Everett MG Christley RM. Recent network evolution increases the potential for large epidemics in the British cattle population. J R Soc Interf. (2007) 4:669–74. 10.1098/rsif.2007.0214 17284415 PMC2373390

[18] Natale F Giovannini A Savini L Palma D Possenti L Fiore G. Network analysis of Italian cattle trade patterns and evaluation of risks for potential disease spread. Prevent Vet Med. (2009) 92:341–50. 10.1016/j.prevetmed.2009.08.026 19775765

[19] Volkova VV Howey R Savill NJ Woolhouse MEJ. Sheep movement networks and transmission of infectious diseases. PLoS ONE. (2010) 5:e11185. 10.1371/journal.pone.0011185 20567504 PMC2887355

[20] Aznar MN Stevenson MA Zarich L Leon EA. Analysis of cattle movements in Argentina. Prevent Vet Med. (2005). 98:119–27. 10.1016/j.prevetmed.2010.11.004 21122931

[21] Nöremark M Hakansson N Sternberg Lewerin S Lindberg A Jonsson A. Network analysis of cattle and pig movements in Sweden: measures relevant for disease control and risk-based surveillance. Prevent Vet Med. (2011) 99:78–90. 10.1016/j.prevetmed.2010.12.009 21288583

[22] Mweu MM Fournié G Halasa T Toft N Nielsen SS Temporal characterization of the network of Danish cattle movements and its implication for disease control: 2000-2009. Prevent Vet Med. (2013) 110:379–87. 10.1016/j.prevetmed.2013.02.015 23473852

[23] Brooks-Pollock E Roberts GO Keeling MJ. A dynamic model of bovine tuberculosis spread and control in Great Britain. Nature. (2014) 511:228–31. 10.1038/nature13529 25008532

[24] Vanderwaal KL Picasso C Enns EA Craft ME Alvarez J Fernandez F. Network analysis of cattle movements in Uruguay: quantifying heterogeneity for risk-based disease surveillance and control. Prevent Vet Med. (2016) 123:12–22. 10.1016/j.prevetmed.2015.12.003 26708252

[25] Corner LA Pfeiffer D Morris RS. Social network analysis of *Mycobacterium bovis* transmission among captive brushtail possums (*Trichisurus Vulpecula*). Prevent Vet Med. (2003) 59:147–67. 10.1016/S0167-5877(03)00075-8 12809760

[26] Christley RM Pinchbeck GL Bowers RG Clancy D French NP Bennett R. Infection in social networks: using network analysis to identify high-risk individuals. Am J Epidemiol. (2003) 162:1024–31. 10.1093/aje/kwi308 16177140

[27] Watts DJ Six Degrees: The Science of a Connected Age. W. W. Norton and Company (2003).

[28] De Nooy W Mrvar A Batagelj V Exploratory Network Analysis with Pajek. Cambrigde New York, NY: University Press (2005). p. 362.

[29] Jackson MO Social and Economic Networks. Princeton, NJ: Princeton University Press (2010). 10.2307/j.ctvcm4gh1

[30] Keeling MJ Eames KTD Network and epidemic models. J R Soc Interf. (2005) 2:195–307. 10.1098/rsif.2005.0051 PMC1578276 16849187

[31] Martínez-López B Perez AM Sánchez-Vizcaíno JM. Combined application of social network and cluster detection analyses for temporal-spatial characterization of animal movements in Salamanca, Spain. Prevent Vet Med. (2009) 91:29–38. 10.1016/j.prevetmed.2009.05.007 19500865

[32] Newman MEJ Networks: An Introduction. New York, NY: Oxford University Press (2010).

[33] IBGE Produção da Pecuária Municipal. (2016). Available online at: http://biblioteca.ibge.gov.br/visualizacao/periodicos/84/ppm_2015_v43_br.pdf (accessed 12, Jul. 2017).

[34] R Development Core Team. R: A Language and Environment for Statistical Computing. R Foundation for Statistical Computing, Vienna, Austria (2019). Available online at: http://www.R-project.org.

[35] Wickham H François R Henry L Müller K dplyr: A Grammar of Data Manipulation. R package version 0.8.1 (2019). Available online at: https://cran.r-project.org/web/packages/dplyr/index.html.

[36] Csárdi G igraph: Network Analysis and Visualization. R package version 1.2.4.1 (2019). Available online at: https://cran.r-project.org/web/packages/igraph/index.html.

[37] Negreiros RL Amaku M Dias RA Ferreira F Cavalléro JCM Ferreira Neto JS Spatial clustering analysis of the foot-and-mouth disease outbreaks in Mato Grosso do Sul state, Brazil. Ciência Rural. (2009) 39:2609–13. 10.1590/S0103-84782009005000203

[38] Garcia DCC Mcmanus CM De Melo CB Impactos do surto de febre aftosa de 2005 sobre as exportações de carne bovina Brasileira. Ciência Animal Brasileira. (2015) 16:525–37. 10.1590/1089-6891v16i426158

[39] Santos DV Silva GS Weber EJ Hasenack H Groff FHS Todeschini B. Identification of foot and mouth disease risk areas using a multi-criteria analysis approach. PLoS ONE. (2017) 12:e0178464. 10.1371/journal.pone.0178464 28552973 PMC5446179

[40] Lal Dutta B Ezanno P Vergu E Characteristics of the spatio-temporal network of cattle movements in France over a 5-year period. Prevent Vet Med. (2014) 117:79–94. 10.1016/j.prevetmed.2014.09.005 25287322

[41] Correr GN Crespolini GN Ribeiro GG De Zen S Estudo do Abate Bovino no Brasil: Uma Proposta Metodológica. 56° Congresso da Sociedade Brasileira de Economia, Administração e Sociologia Rural (2018).

[42] Garner MG Beckett SD. Modelling the spread of foot-and-mouth disease in Australia. Austral Vet J. (2005) 83:758–66. 10.1111/j.1751-0813.2005.tb11589.x 16395942

[43] Gale SB Miller GY Eshelman CE Wells SJ. Epidemic simulation of a foot and mouth disease outbreak in Minnesota. Revue Sci Tech. (2015) 34:895–905. 10.20506/rst.34.3.2404 27044160

